# Prenatal Exposure to Butylbenzyl Phthalate and Early Eczema in an Urban Cohort

**DOI:** 10.1289/ehp.1104544

**Published:** 2012-06-26

**Authors:** Allan C. Just, Robin M. Whyatt, Matthew S. Perzanowski, Antonia M. Calafat, Frederica P. Perera, Inge F. Goldstein, Qixuan Chen, Andrew G. Rundle, Rachel L. Miller

**Affiliations:** 1 Columbia Center for Children’s Environmental Health, Department of Environmental Health Sciences, Mailman School of Public Health, Columbia University, New York, New York, USA; 2 National Center for Environmental Health, Centers for Disease Control and Prevention, Atlanta, Georgia, USA; 3 Department of Epidemiology, and; 4 Department of Biostatistics, Mailman School of Public Health, Columbia University, New York, New York, USA; 5 Division of Pulmonary, Allergy, Critical Care, Department of Medicine, Columbia University College of Physicians and Surgeons, New York, New York, USA

**Keywords:** butylbenzyl phthalate, eczema, plasticizers

## Abstract

Background: Recent cross-sectional studies suggest a link between butylbenzyl phthalate (BBzP) in house dust and childhood eczema.

Objectives: We aimed to evaluate whether concentrations of monobenzyl phthalate (MBzP), the main BBzP metabolite in urine, during pregnancy are associated prospectively with eczema in young children, and whether this association varies by the child’s sensitization to indoor allergens or serological evidence of any allergies.

Methods: MBzP was measured in spot urine samples during the third trimester of pregnancy from 407 African-American and Dominican women residing in New York City in 1999–2006. Repeated questionnaires asked mothers whether their doctor ever said their child had eczema. Child blood samples at 24, 36, and 60 months of age were analyzed for total, anti-cockroach, dust mite, and mouse IgE. Relative risks (RR) were estimated with multivariable modified Poisson regression. Analyses included a multinomial logistic regression model for early- and late-onset eczema versus no eczema through 60 months of age.

Results: MBzP was detected in > 99% of samples (geometric mean = 13.6; interquartile range: 5.7–31.1 ng/mL). By 24 months, 30% of children developed eczema, with the proportion higher among African Americans (48%) than among Dominicans (21%) (*p* < 0.001). An interquartile range increase in log MBzP concentration was associated positively with early-onset eczema (RR = 1.52 for eczema by 24 months; 95% confidence interval: 1.21, 1.91, *p* = 0.0003, *n* = 113 reporting eczema/376 total sample), adjusting for urine specific gravity, sex, and race/ethnicity. MBzP was not associated with allergic sensitization, nor did seroatopy modify consistently the MBzP and eczema association.

Conclusions: Prenatal exposure to BBzP may influence the risk of developing eczema in early childhood.

Eczema, a type of persistent itchy skin rash, in children may be an early manifestation of allergic disease (Leung et al. 2004). Although exposure to allergens may be important in the development of eczema, exposure to other environmental agents has been implicated as well. For example, in cross-sectional studies, exposure to environmental tobacco smoke, airborne propylene glycol, glycol ether, and residential renovation around the time of birth have been associated with eczema in young children (Choi et al. 2010; Herbarth et al. 2006; Kramer et al. 2004). Results from a 2003 U.S. national survey suggest that living in urban areas, African-American race, higher parental education, and child-care attendance are associated with an increased prevalence of childhood eczema (Shaw et al. 2011). Further, not all childhood eczema as defined by the World Allergy Organization (Johansson et al. 2004) is allergic in origin. Either allergic or nonatopic eczema (i.e., intrinsic or nonallergic eczema/dermatitis) may develop. The two phenotypes differ somewhat in their risk factors and in their immunopathogenesis (Kusel et al. 2005).

Some phthalates are added to plastics to increase flexibility and are common components of many plastic household products; others, such as butylbenzyl phthalate (BBzP), are added to vinyl floorings. BBzP is found frequently in house dust and indoor air (Adibi et al. 2008; Kolarik et al. 2008a). Monobenzyl phthalate (MBzP), the main BBzP metabolite, was detected in greater than 97% of urine samples in the U.S. 1999–2000 National Health and Nutrition Examination Survey (NHANES) (Silva et al. 2004) and detected in a pilot study (*n* = 11) from amniotic fluid in Germany (Wittassek et al. 2009). Although diet is believed generally to be the largest contributor to BBzP exposure in adults (Wormuth et al. 2006), we reported that concentrations of BBzP collected in 48-hr personal air samples during pregnancy from participants in the Columbia Center for Children’s Environmental Health (CCCEH) were correlated with concentrations of MBzP in spot urine samples (*r*_S_ = 0.48, *p* < 0.05, *n =* 62) (Adibi et al. 2008). Hence, nondietary routes, including inhalation and dust exposure through skin and ingestion, also may be important.

In addition, concentrations of BBzP in bedroom dust in a Swedish study were significantly higher among children 3–8 years of age with physician-confirmed persistent eczema and rhinitis (*n* = 198) versus healthy children (*n* = 202) with a positive dose–response trend in the odds ratio (OR) across quartiles of exposure. Polyvinyl chloride (PVC) flooring, considered an important source of phthalate plasticizers in the home, also was associated with persistent allergic symptoms (Bornehag et al. 2004). Consistent findings were reported by a study of 100 cases and 77 nonsymptomatic control children 2–7 years of age in Bulgaria. BBzP concentrations were higher in the house dust of children with wheeze, rhinitis, or eczema in the preceding 12 months, but the difference was not statistically significant (mean = 0.38 mg/g dust vs. 0.32 mg/g dust, *p* = 0.35) (Kolarik et al. 2008b). Although these findings suggest a role of BBzP in the development of eczema, the use of urinary biomarkers offers a more direct measure of individual exposure than house dust by integrating ingestion, inhalation, and dermal absorption. In addition, longitudinal studies may offer further insight into the timing of exposure relative to disease onset.

The purpose of this study was to investigate whether prenatal measures of BBzP are associated with eczema and sensitization to common indoor aeroallergens in early childhood in an urban birth cohort. We hypothesized that prenatal BBzP would be associated with increased risk of eczema and elevated indoor allergen specific and total immunoglobulin (Ig) E.

## Methods

As part of the CCCEH birth cohort, 727 nonsmoking pregnant Dominican and African-American women were fully enrolled during the third trimester, and mother–child pairs were followed prospectively (Perera et al. 2003). Participant mothers for the present analysis (*n =* 407) had prenatal urinary phthalate metabolites measured and responded to at least one questionnaire on eczema when the child age was between 3 and 60 months. The study was approved by the institutional review boards (IRBs) of Columbia University and the Centers for Disease Control and Prevention (CDC). The mother provided informed signed consent at time of enrollment in accordance with IRB requirements.

Spot urine samples were collected during the third trimester [mean gestational age, 34 weeks, interquartile range (IQR) 32–36 weeks] from 1999 through 2006. Samples were stored at –80°C until overnight shipment with dry ice to the CDC, where they were stored at –70°C until being analyzed for metabolites of BBzP and several other phthalates at the National Center for Environmental Health (Adibi et al. 2008; Kato et al. 2005). A correction factor of 0.72 was applied to the MBzP concentrations to adjust for previous overestimations of the analytic standards (CDC 2012). Two metabolites of other phthalates, mono-*n*-butyl phthalate and mono(2-ethyl-5-hydroxyhexyl) phthalate, known to correlate moderately with MBzP, also were considered in two-metabolite co-pollutant models. The specific gravity of the urine was measured after thaw using a handheld refractometer (PAL 10-S; Atago USA, Bellevue, WA).

Eczema was assessed by questionnaires administered to the mother by telephone and in-person at repeated visits by asking “Has your doctor ever said that your child has eczema?” Four questionnaires were administered in each of the first 2 postnatal years and another four questionnaires between child ages 24 and 60 months (for a maximum of 12 questionnaires). Two-thirds of participants answered at least 9 questionnaires over the 12 possible time points. When the child was 60 months, the International Study of Asthma and Allergies in Childhood (ISAAC) eczema module (Asher et al. 1995) also asked “Has your child ever had an itchy rash which was coming and going for at least 6 months?” and among those who responded positively “Has your child had this itchy rash at any time in the last 12 months?”

Sera were collected from the children at 24, 36, and 60 months of age. Because the odds of eczema were higher among children 24–36 months of age who developed IgE responses specifically against cockroach and mouse allergens (but not total IgE), and anti-cockroach IgE correlated closely with total IgE at 24 and 36 months of age (Chang et al. 2010; Donohue et al. 2008), sensitization to indoor allergens was defined as a positive (≥ 0.35 IU/mL) specific IgE to cockroach, dust mite, or mouse in any available sample by ImmunoCAP (Phadia, Uppsala, Sweden). To capture any seroatopy as well, total IgE was measured by either immunoradiometric assay (Diagnostics Products Corp, Los Angeles, CA) or by ImmunoCAP, as described (Donohue et al. 2008).

*Statistical analysis.* We compared descriptive statistics among data subsets with *t*-tests and chi-square tests for continuous and categorical variables, respectively. Urinary concentrations of MBzP were log transformed before analysis. Specific gravity was retained as a covariate in all models to account for variation in urinary dilution (Barr et al. 2005). Sex and race/ethnicity were included as covariates in all models as *a priori* potential confounders. A series of other potential confounders [prenatal exposure to environmental tobacco smoke, and maternal attributes (age, education, marital status, self-report of asthma, log total IgE)] were tested sequentially for inclusion in the final model based on a > 10% change in the parameter estimate for the association of MBzP and early-onset eczema. One participant with a concentration of MBzP below the limit of detection (0.22 ng/mL; specific gravity 1.005) was excluded from further analysis because imputing a concentration of half the limit of detection and subsequent log transformation of the predictor created an influential point in the regression. Sensitivity analysis showed that parameter estimates excluding this sample did not differ from those in which 1 ng/mL was added to all concentrations before log transformation.

We used a modified Poisson regression to generate relative risk (RR) and variance estimates for dichotomous outcomes (i.e., eczema, sensitization to indoor allergens) (Zou 2004). Eczema was reclassified as early-onset eczema if it was reported on any questionnaire through 24 months of age. This combined variable for early-onset eczema was restricted to children with at least one of four questionnaires in the first year, and one of four in the second year (*n =* 376). Eczema was reclassified as late-onset eczema if the first report of ever eczema was reported between age 24 months and 60 months among participants who completed at least one of four questionnaires in the first year, one of four in the second year, and one of four in the third through fifth year. A multinomial logistic regression was used to estimate associations between MBzP and early-onset eczema or late-onset eczema, with no eczema through age 60 months as the referent outcome, among participants who remained enrolled through 60 months (*n =* 339).

Data on indoor allergen sensitization using specific IgE were available for 94% of children in the early-onset eczema analysis (*n =* 355) and total IgE at 60 months of age was available for 78% (*n* = 295). Although the RR regression was a multiplicative model, potential interactions between risk factors were tested as a departure from additivity using the relative excess risk due to interaction (RERI) (Rothman et al. 2008), with 95% confidence intervals (CIs) from percentiles of 10,000 bootstrap samples (Knol et al. 2007). Linear regression was used for modeling log-transformed total IgE as a continuous outcome.

Statistical analyses were conducted using R statistical software (version 2.13.0; R Development Core Team, Vienna, Austria) where *p* < 0.05 was considered statistically significant.

## Results

*Cohort characteristics.* Participants included in analyses did not differ in major demographic characteristics from the remainder of the CCCEH cohort, except for lower prenatal exposure to environmental tobacco smoke (31% vs. 39%) and sensitization to indoor allergens at 24 months (8% vs. 17%); however, indoor allergen-specific sensitization did not differ at 36 or 60 months or when measures from any of the three ages (24, 36, 60 months) among available samples for specific IgE were combined and comparisons between the two groups were reanalyzed (Table 1). MBzP was detected in all except one urine sample, and concentrations ranged widely [geometric mean (GM) = 13.6, IQR: 5.7–31.1 ng/mL]. MBzP concentrations were higher among African American (GM = 18.3; IQR: 8.7–35.4 ng/mL) than Dominican mothers (GM = 11.7, IQR: 4.9–26.4 ng/mL) (*p* < 0.001).

**Table 1:** Characteristics of mother–child pairs included and excluded from analysis.

Characteristic	Participants included^a^ *n* = 407	Participants excluded^b^ *n* = 320	*p*-Value
Mother’s age [mean (IQR)]c	25 (21–29)	25 (21–29)	0.13
Mother’s ethnicity (%)	0.46
African American	34	37
Dominican	66	63
Mother completed high school, GED, or greater (%)	63	66	0.45
Never married (%)	66	66	1.00
Prenatal ETS exposure (%)d	31	39	0.03
Maternal asthma (%)	25	19	0.10
Maternal total IgE > 80 IU/mL (%)	36	34	0.74
Child’s sex (% male)	48	48	1.00
Child’s indoor allergic sensitization (%)e
At 24 months	8	17	0.01
At 36 months	14	15	0.82
At 60 months	26	24	0.79
At any of the three ages (24, 36, or 60 months)	26	29	0.44
Abbreviations: ETS, environmental tobacco smoke; GED, General Educational Development; GM, geometric mean. aMissing: maternal total IgE (n = 143), child’s indoor allergic sensitization at 24 months (n = 136), 36 months (n = 116), 60 months (n = 93), combined time points (n = 40). bMissing: education (n = 14), never married (n = 6), maternal total IgE (n = 129), child’s indoor allergic sensitization at 24 months (n = 143), 36 months (n = 153), 60 months (n = 147), at any of the three ages (i.e., measures from 24, 36, or 60 months among children with any available specific IgE levels; n = 95). cAssessed with Wilcoxon rank-sum test. dPrenatal ETS exposed if maternal or cord blood cotinine concentration ≥ 15 ng/mL (available for n = 391 and n = 306, respectively) or report of a smoker in the home on prenatal questionnaire. eIndoor allergic sensitization defined as ≥ 0.35 IU/mL specific IgE to cockroach, dust mite, or mouse.						

**Figure 1:**
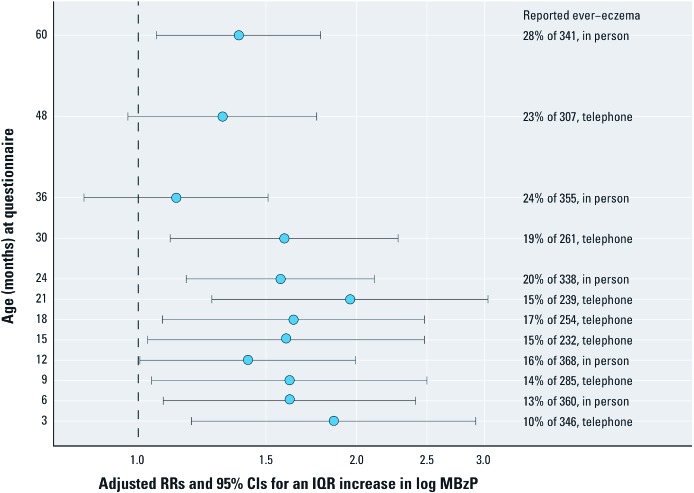
Relative risk estimates of child ever-eczema for an IQR increase in prenatal log MBzP urinary concentration (IQR is 1.7 log units, 5.7–31.1 ng/mL), adjusting for specific gravity, race/ethnicity, and sex from separate modified Poisson regression models using questionnaire data collected in person or by telephone interview at 12 different ages.

*Eczema characteristics.* By child age 24 months, 30% of mothers had reported at least once that their doctor had ever said that their child had eczema, with a higher proportion among African-American (48%, *n* = 62/129) than Dominican (21%, *n* = 51/247) children. The proportion of Dominican mothers reporting eczema in their children was comparable among mothers completing questionnaires in English versus Spanish. There was no significant difference in the proportion of children with ever-eczema by child sex overall (32% female, 28% male, *p* = 0.37) or after stratifying by race/ethnicity (data not shown). The proportion of children with ever eczema generally increased with the child’s age, from 10% at 3 months to 28% at 60 months (Figure 1). However, women were sometimes inconsistent in their report of their child’s eczema. Among those who had a positive report of ever-eczema (at any of the 12 questionnaire time points), 59% of subsequent questionnaires were also positive.

*MBzP and eczema.* Adjusted RRs of child ever-eczema for an IQR increase in prenatal MBzP urinary concentrations at each of 12 questionnaire time points were consistently positive with larger magnitudes at the earlier ages (Figure 1). Using a reclassified definition of early onset eczema, an IQR increase in log MBzP was associated positively with any report of eczema by 24 months (RR = 1.52; 95% CI: 1.21, 1.91; *p* < 0.001, *n =* 113/376) after adjusting for specific gravity, sex, and race/ethnicity. The parameter estimate for child’s sex was not significant (RR = 0.82 for males vs. females; 95% CI: 0.60, 1.10). Adjusting for prenatal exposure to environmental tobacco smoke, or maternal attributes (age, education, marital status, self-report of asthma, log total IgE) changed the main effect of MBzP by < 2%, and these variables were not retained in the adjusted model. ORs from a three-outcome multinomial logistic regression model were comparable for ever-eczema among inconsistent reporters (who said no ever-eczema after having said yes ever-eczema) (OR = 1.98; 95% CI: 1.23, 3.17; *n* = 63) and consistent reporters (OR = 2.02; 95% CI: 1.19, 3.43; *n* = 50) compared with those who never reported eczema (*n* = 263). A second sensitivity analysis defined the outcome for consistent reporters to those with three or more consecutive positive reports among those with earlier first report of eczema (OR = 2.25; 95% CI: 1.22, 4.13 vs. never eczema, and OR = 1.90; 95% CI: 1.23, 2.95 for inconsistent or < 3 positive reports vs. never eczema). To determine whether the association between an IQR increase in prenatal log MBzP concentrations and eczema was greater among children with early onset (by 24 months) versus late-onset eczema, multinomial analyses were performed after reclassifying eczema as early (29%, *n =* 100), late (13%, *n =* 43), or none (58%, *n =* 196). MBzP concentration was associated with early onset eczema (adjusted OR = 1.91; 95% CI: 1.23, 2.97). There was no association between MBzP and late onset eczema (adjusted OR = 0.90; 95% CI: 0.51, 1.58) for an IQR change in log MBzP from the multinomial logistic regression analysis. Moreover, there was no association in a modified Poisson regression model between MBzP concentration and the report at 60 months of “itchy rash at any time in the past 12 months that was coming and going for at least 6 months” (RR = 1.23; 95% CI: 0.81, 1.88; *n =* 56/341).

**Figure 2:**
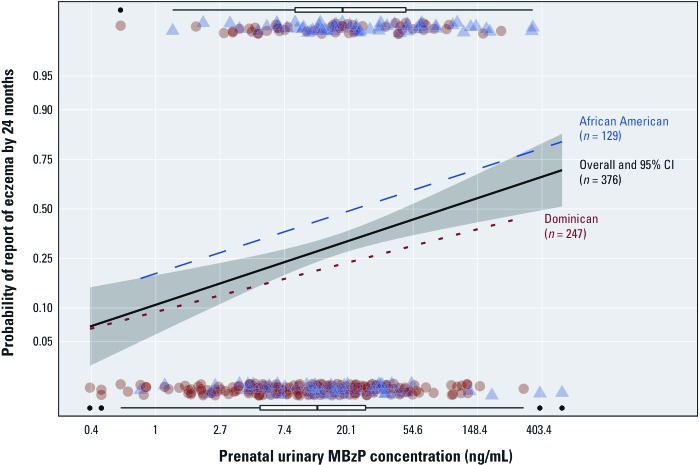
Association between prenatal urinary concentration of MBzP and report of eczema by 24 months from a multivariable logistic regression adjusted with mean specific gravity and sex. The *y*-axis is shown on the logit scale and labeled with the corresponding predicted probabilities. The *x*-axis is shown on a log scale and labeled with the corresponding concentrations. The overall cohort model is shown (black) with 95% CI (gray); the exposure distribution for all participants reporting (top) and not reporting (bottom) eczema is shown with horizontal box plots. The ethnic subsets are shown in dashed and dotted lines for African Americans and Dominicans, with individual observations shown at their urinary concentration as triangles and circles, respectively. Although the baseline probabilities differ in the two subsets, the overall association remains positive, with increasing urinary concentrations of MBzP.

*MBzP and eczema by ethnicity.* Because previously we reported that eczema may vary by race/ethnicity (Donohue et al. 2008), we sought to understand better whether the association between phthalates and eczema was modified by race/ethnicity. Maternal African-American race versus Dominican ethnicity was a significant predictor of report of eczema by 24 months (RR = 2.09; 95% CI: 1.53, 2.86; *p* < 0.001) after adjusting for MBzP concentration, specific gravity, and sex. There was a consistently higher probability of eczema for African Americans across the range of urinary concentrations of MBzP. Both ethnic groups had similar slopes for the exposure–response curve in stratified analysis, indicating a lack of multiplicative interaction (Figure 2). However, there was a nonsignificant greater than additive interaction between African American ethnicity and each IQR increase in MBzP urinary concentration (RR African American 2.47; RR IQR MBzP 1.60; joint RR 3.59; RERI 0.52; bootstrap 95% CI: –0.11, 1.31).

*MBzP, eczema, and allergic sensitization.* A total of 26% (*n =* 91/355) of children were classified as sensitized to indoor aeroallergens at 24, 36, or 60 months of age. Among children with eczema by 24 months, 29% (*n =* 31/106) were sensitized compared with 24% among children without eczema (*n =* 60/249) (*p* = 0.31). Children with eczema by 24 months on average had higher total IgE levels at 60 months of age than children without eczema, regardless of whether they were defined as nonsensitized (GM = 39 IU/mL, *n =* 67 vs. 24 IU/mL, *n =* 152; *p* = 0.016) or sensitized to indoor aeroallergens (GM = 207 IU/mL, *n =* 22 vs. 107 IU/mL, *n =* 50; *p* = 0.056).

There was no observed association between an IQR increase in prenatal log MBzP urinary concentration and sensitization to indoor aeroallergens (RR = 0.89; 95% CI: 0.68, 1.16, *n =* 355), adjusting for specific gravity, race/ethnicity, and sex. Similarly, there was no observed association between an IQR increase in log MBzP concentration and log total IgE at 60 months in adjusted analyses (β = –0.14; 95% CI: –0.41, 0.13, *n =* 306). We also examined whether the association between prenatal MBzP urinary concentration and eczema differed by sensitization to any of three indoor aeroallergens. There was no significant interaction between sensitization to indoor allergens and higher MBzP concentration in predicting eczema using the RERI to test for departures from additivity (RR sensitization 1.19; RR IQR MBzP 1.54; joint RR 1.87; RERI 0.14; bootstrap 95% CI: –0.34, 0.42). Retesting the RERI while examining interactions between any seroatopy (i.e., using total IgE as a continuous variable) and higher MBzP concentration in predicting eczema found a slightly greater than additive effect of MBzP and log total IgE at 60 months of age (RR log total IgE 1.18; RR IQR MBzP 1.46; joint RR 1.72; RERI: 0.07; 95% CI: 0.01, 0.46; *n* = 295).

*Other phthalates and eczema.* Although urinary concentrations of the metabolites of several other phthalates correlated moderately with MBzP, in adjusted models with both prenatal MBzP and mono-*n*-butyl phthalate (MnBP; a major metabolite of di-*n*-butyl phthalate and a minor metabolite of BBzP) or mono(2-ethyl-5-hydroxyhexyl) phthalate [MEHHP; a metabolite of di(2-ethylhexyl) phthalate (DEHP)] urinary concentrations, the estimate for MBzP was changed by < 10%. In these two-metabolite models, only MBzP was a significant predictor of report of eczema by 24 months (RR per log unit MnBP = 1.13; 95% CI: 0.89, 1.43; *n* = 376; RR per log unit MEHHP = 1.06; 95% CI: 0.75, 1.51; *n* = 376) adjusting for log MBzP, specific gravity, race/ethnicity, and sex.

## Discussion

We report here a novel association between prenatal urinary concentrations of MBzP, the main metabolite of BBzP, and the subsequent report of childhood eczema among African-American and Dominican children in New York City. The association appeared to be primarily among those with early-onset eczema, reported by 24 months of age. Although the proportion of children with eczema was higher in African Americans than in Dominicans, in both groups there was a similar positive association between prenatal urinary concentrations of MBzP and early-onset eczema. No association was observed between prenatal urinary concentration of MBzP and sensitization to three indoor allergens or total IgE. The association of MBzP and eczema was not modified by sensitization to indoor allergens and only an isolated finding of an additive interaction for MBzP and total IgE at 60 months of age in predicting eczema was observed. The estimated combined effect of an IQR increase in log MBzP and a 1–log unit increase in total IgE at 60 months of age was slightly greater than expected if the effects of MBzP alone and log total IgE at 60 months of age alone were added. These prospective results extend previous cross-sectional results nested in a Swedish cohort linking house dust concentrations of BBzP and eczema in a dose–response manner (*n* = 115 with eczema and *n* = 177 controls) (Bornehag et al. 2004). Although Swedish case children 3–8 years of age with physician-diagnosed eczema had statistically significantly higher BBzP concentration in their bedroom dust than controls, consistent with the findings here, there was no difference in BBzP concentration between atopic and nonatopic cases (Bornehag et al. 2004).

One particular strength of the present study is the use of a urinary biomarker of phthalate exposure that has been shown previously in this population to have good reproducibility over late pregnancy [intraclass correlation coefficient (ICC) = 0.66 for MBzP sampled 2–4 times over 6 weeks] (Adibi et al. 2008). In addition, the prospective birth cohort design allows the measure of prenatal exposure to precede the development and subsequent report of children’s health outcomes. It also has enabled us to evaluate the association between prenatal exposure to BBzP and early- versus late-onset eczema based on the age at first report. When questionnaires asked at different ages were analyzed with separate models, associations between prenatal urinary concentrations of MBzP and eczema had larger effect size estimates on questionnaires collected at earlier ages than those after 24 months. In the multinomial analysis, MBzP concentration was associated with early but not late onset eczema (first report after 24 months) relative to those in the no-eczema group.

Given the known association between eczema and atopy, and between eczema and cockroach and mouse IgE in the CCCEH cohort (Donohue et al. 2008), we also hypothesized that exposure to BBzP would be associated with sensitization to the three most common indoor aeroallergens. Experimental evidence, at least with other phthalates, supported this approach. For example, human peripheral blood mononuclear cells incubated with DEHP, or its hydrolytic metabolite mono(2-ethylhexyl) phthalate (MEHP), produced greater proallergic cytokine levels and histamine release [reviewed by Kwak et al. (2009)]. In addition, prenatal and neonatal exposure of mice to DEHP induced an atopic dermatitis-like phenotype in dust mite–sensitized offspring and up-regulated the expression of the proallergic chemokine eotaxin (Yanagisawa et al. 2008). Interestingly, another phthalate, di-*n*-butyl phthalate, up-regulated skin hypersensitivity reactions via thymic stromal lymphopoietin, a cytokine known to activate the maturation of dendritic cells and recently associated with eczema, in mice (Larson et al. 2010; Shigeno et al. 2009).

However, our findings suggest that exposure to BBzP may increase the risk of eczema through a nonallergic mechanism. Although eczema is considered frequently an early indicator of IgE-mediated allergic diseases (Leung et al. 2004), eczema among non-atopic children also is prevalent. Two-thirds of children with eczema are nonatopic in population-based studies (Flohr et al. 2004). In NHANES 2005–2006, 68% of children 6–10 years of age with reported eczema did not have sensitization to indoor allergens, and 36% did not have elevated (i.e., > 50 IU/mL) total IgE levels (NCHS 2008), compared with 71% and 40% respectively at 60 months of age in this African-American and Dominican cohort. Further, evidence suggests that immunopathogenesis of eczema can be triggered by mechanisms both unrelated and related to allergic sensitization. For example, transgenic mice overexpressing interleukin (IL)–31 develop eczema-like skin lesions with features characteristic of nonatopic dermatitis (lack of increased IgE) (Dillon et al. 2004). However, additional studies involving stimulation of human epidermal keritonocyte cell lines with human IL-31 induced the expression of several proallergic chemokines, including TARC/CCL17 (thymus- and activation-regulated chemokine) and MDC (macrophage-derived chemokine)/CCL22 that are important to the recruitment of inflammatory cells to the skin (Dillon et al. 2004). The presence of single nucleotide polymorphisms in IL-31 in analyses combining data from three independent European study populations was linked to nonatopic, and not atopic, eczema (Schulz et al. 2007). In an *in vitro* study of the human epithelial cell line A549, several phthalate metabolites, including MEHP and mono-*n*-octyl phthalate, but not MBzP, stimulated the production of the nonallergic, proinflammatory cytokine IL-8 (Jepsen et al. 2004). Finally, in an *in vitro* study, culture of Cos-1 cell lines with BBzP upon transfection with either the peroxisome proliferator-activated receptors (PPAR)α or PPARγ-expressing plasmid increased the transcriptional activity of both PPARα and PPARγ (Hurst and Waxman 2003). Mice deficient in the *PPAR*α gene are more likely to develop eczema-like skin lesions upon antigen sensitization (Staumont-Salle et al. 2008), suggesting that the expression of this gene is more likely to protect against, rather than induce, eczema.

The association between eczema and phthalate exposure may vary by race/ethnicity. The joint effect of an IQR increase in MBzP urinary concentration and African-American race/ethnicity resulted in a RR estimate that was 0.54 higher (bootstrap 95% CI on RERI: –0.07, 1.39) than the expected sum of the main effects estimated in the absence of interaction, although this additive interaction did not reach statistical significance. We speculate that differences in dietary patterns as well as differences in the prevalence of indoor materials, such as vinyl flooring made with BBzP, may contribute to the observed higher urinary concentrations of MBzP among African Americans. Differences in eczema prevalence by race/ethnicity have been reported in the 2003 National Survey of Children’s Health; 9.7% of white-only and 15.9% of black-only households reported eczema in the previous year for children < 18 years of age (Shaw et al. 2011). Race remained a significant predictor after controlling for a number of potential confounders also associated with higher eczema such as young child age, higher level of education, higher family income, and urban residence (Shaw et al. 2011). Among children 6–10 years of age included in the NHANES 2005–2006 with serum IgE measures, 4% of Mexican-American respondents (*n* = 12/267), and 22% of African-American respondents (*n* = 45/201) said yes to “Has a doctor or other health professional ever told you that __ has eczema?” (National Center for Health Statistics 2008). The subset of Mexican-American participants in NHANES may not be an appropriate comparison population for Dominican-American families in New York City; only 3.3% of NHANES respondents were classified as “Hispanic–Other Hispanic,” thus limiting the ability to compare the NHANES estimate with that for our Dominican population in New York City. In addition, the NHANES estimate was for an older group of children.

We acknowledge several limitations. There was no assessment of postnatal exposure to phthalates. Child eczema was assessed via questionnaire and therefore subject to a lack of standardization, misclassification, or recall bias. The lack of consistent reporting of eczema after the first report of ever-eczema may indicate some misclassification. However, our query “Has your doctor ever said that your child has eczema?” has been validated by physician diagnosis in a study of Oregon school children (Laughter et al. 2000). Because the question specifically asked about doctor-diagnosed eczema, misclassification also could be associated with access to health care, although it is unclear how access to health care could be related to phthalate exposure. Also there is no reason to believe that any misclassification would be differential by phthalate exposure and thus should only bias the effect estimates toward the null. Furthermore, the main association did not differ between consistent and inconsistent reporters of ever-eczema in the sensitivity analyses. Some of the differences in the report of eczema by ethnicity may partially reflect differences in the understanding of what constitutes “eczema,” although there were no differences in report between English- and Spanish-speaking Dominican participants. In addition, the contribution of food allergy to MBzP-associated early eczema was not assessed, and the higher total IgE among those with eczema than those without eczema suggests that sensitization to unmeasured allergens might be associated with eczema here. A larger suite of specific IgEs (anti-cockroach, dust mite, mouse, cat, dog, tree, grass, ragweed, and mold) were tested at 7 years of age in this cohort and were available for comparison with 197 children in this analysis. Among these children, 25% (50/197) were classified as sensitized to indoor allergens (cockroach, dust mite, or mouse) by 5 years, whereas at 7 years this proportion increased to 34% (67/197). Expanding to a definition of any indoor or outdoor IgEs at 7 years of age, 46% (90/197) would be classified as seroatopic. Thus, in a subset with data through age 7 years, the use of specific IgE to cockroach, dust mite, or mouse to characterize sensitization to indoor allergens underestimates the proportion of children with any common indoor or outdoor allergic sensitization by about one-quarter (23/90). We also cannot rule out that children classified as not sensitized to indoor allergens may later develop sensitization. Although sensitization to indoor allergens is not the same as any seroatopy, the consistency between results using sensitization to any of three indoor specific IgEs and continuous total IgE concentration suggests this is not a major concern. Rates of sensitization differed at 24 months, although not at 36 or 60 months, among participants included versus excluded from the analysis, raising a small possibility of bias. However, baseline characteristics of participants included versus excluded from analysis were otherwise very similar, likely because 94% of those excluded lacked the prenatal urine sample for phthalates added after the initiation of the cohort and unlikely to be systematically associated with exposure or outcomes.

## Conclusions

Using a longitudinal urban U.S. birth cohort, we found novel results that suggest that MBzP urinary concentrations measured prenatally are associated with early-onset eczema. This finding seemed unrelated to sensitization to common indoor allergens or total IgE. Future intervention studies may test whether early-life exposure to BBzP can be reduced and whether this leads to a reduction in eczema, a very common childhood disease.
